# Biphasic positive airway pressure minimizes biological impact on lung tissue in mild acute lung injury independent of etiology

**DOI:** 10.1186/cc13051

**Published:** 2013-10-08

**Authors:** Felipe Saddy, Lillian Moraes, Cintia Lourenço Santos, Gisele Pena Oliveira, Fernanda Ferreira Cruz, Marcelo Marcos Morales, Vera Luiza Capelozzi, Marcelo Gama de Abreu, Cristiane Souza Nascimento Baez Garcia, Paolo Pelosi, Patricia Rieken Macêdo Rocco

**Affiliations:** 1 Laboratory of Pulmonary Investigation, Carlos Chagas Filho Biophysics Institute, Federal University of Rio de Janeiro, Centro de Ciências da Saúde, Av Carlos Chagas Filho, s/n, Bloco G-014, Ilha do Fundão, 21941-902 Rio de Janeiro, RJ, Brazil; 2 Hospital Procardíaco, Rua General Polidoro, 192, Botafogo, 22280-003 Rio de Janeiro, RJ, Brazil; 3 Hospital Copa D’Or, Rua Figueiredo Magalhães, 875, Copacabana, 22031-011 Rio de Janeiro, RJ, Brazil; 4 Laboratory of Experimental Surgery, Faculty of Medicine, Federal University of Rio de Janeiro, Av Professor Rodolpho Paulo Rocco, 225, Ilha do Fundão, 21941-913 Rio de Janeiro, RJ, Brazil; 5 Laboratory of Cellular and Molecular Physiology, Carlos Chagas Filho Biophysics Institute, Federal University of Rio de Janeiro, Av Carlos Chagas Filho, s/n, Bloco G2-048, Ilha do Fundão, 21941-902 Rio de Janeiro, RJ, Brazil; 6 Department of Pathology, School of Medicine, University of São Paulo, Av Doutor Arnaldo, 455, 01246-903 São Paulo, SP, Brazil; 7 Pulmonary Engineering Group, Department of Anesthesiology and Intensive Care Therapy, University Hospital Carl Gustav Carus, Dresden University of Technology, Fetschertsrasse 74, D-01307 Dresden, Germany; 8 Rio de Janeiro Federal Institute of Education, Science and Technology, Rua Carlos Wenceslau, no. 343, Realengo, 21715-000 Rio de Janeiro, RJ, Brazil; 9 IRCCS AOU San Martino-IST, Department of Surgical Sciences and Integrated Diagnostics, University of Genoa, Largo Rosanna Benzi 8, I-16132 Genoa, Italy

## Abstract

**Introduction:**

Biphasic positive airway pressure (BIVENT) is a partial support mode that employs pressure-controlled, time-cycled ventilation set at two levels of continuous positive airway pressure with unrestricted spontaneous breathing. BIVENT can modulate inspiratory effort by modifying the frequency of controlled breaths. Nevertheless, the optimal amount of inspiratory effort to improve respiratory function while minimizing ventilator-associated lung injury during partial ventilatory assistance has not been determined. Furthermore, it is unclear whether the effects of partial ventilatory support depend on acute lung injury (ALI) etiology. This study aimed to investigate the impact of spontaneous and time-cycled control breaths during BIVENT on the lung and diaphragm in experimental pulmonary (p) and extrapulmonary (exp) ALI.

**Methods:**

This was a prospective, randomized, controlled experimental study of 60 adult male Wistar rats. Mild ALI was induced by *Escherichia coli* lipopolysaccharide either intratracheally (ALI_p_) or intraperitoneally (ALI_exp_). After 24 hours, animals were anesthetized and further randomized as follows: (1) pressure-controlled ventilation (PCV) with tidal volume (V_t_) = 6 ml/kg, respiratory rate = 100 breaths/min, PEEP = 5 cmH_2_O, and inspiratory-to-expiratory ratio (I:E) = 1:2; or (2) BIVENT with three spontaneous and time-cycled control breath modes (100, 75, and 50 breaths/min). BIVENT was set with two levels of CPAP (P_high_ = 10 cmH_2_O and P_low_ = 5 cmH_2_O). Inspiratory time was kept constant (T_high_ = 0.3 s).

**Results:**

BIVENT was associated with reduced markers of inflammation, apoptosis, fibrogenesis, and epithelial and endothelial cell damage in lung tissue in both ALI models when compared to PCV. The inspiratory effort during spontaneous breaths increased during BIVENT-50 in both ALI models. In ALI_p_, alveolar collapse was higher in BIVENT-100 than PCV, but decreased during BIVENT-50, and diaphragmatic injury was lower during BIVENT-50 compared to PCV and BIVENT-100. In ALI_exp_, alveolar collapse during BIVENT-100 and BIVENT-75 was comparable to PCV, while decreasing with BIVENT-50, and diaphragmatic injury increased during BIVENT-50.

**Conclusions:**

In mild ALI, BIVENT had a lower biological impact on lung tissue compared to PCV. In contrast, the response of atelectasis and diaphragmatic injury to BIVENT differed according to the rate of spontaneous/controlled breaths and ALI etiology.

## Introduction

Mechanical ventilation with low tidal volume (V_t_), limited inspiratory pressure and positive end-expiratory pressure (PEEP) are commonly used in patients with acute respiratory distress syndrome (ARDS) to minimize alveolar atelectasis and overdistension [1,2]. Protective ventilation is usually associated with controlled modes of mechanical ventilation that may require high-dose sedation and neuromuscular blockade and may also lead to respiratory muscle atrophy, hemodynamic impairment and prolonged weaning [3]. Furthermore, controlled mechanical ventilation may enhance alveolar collapse and inhomogeneity of the lung parenchyma, inducing further lung damage [4].

Partial ventilatory support allows spontaneous breathing efforts during mechanical ventilation, reducing sedation requirements and the need for muscle paralysis, thus minimizing hemodynamic impairment [5] and respiratory muscle dysfunction [3]. In addition, spontaneous breathing has a potentially protective effect on the lung parenchyma resulting from decreased atelectasis and improved ventilation distribution [6]. However, spontaneous breathing activity has the potential to increase transpulmonary pressure (P_L_) and patient–ventilator asynchrony, thereby worsening lung injury and patient outcome in severe ARDS cases [7,8].

Biphasic positive airway pressure (BIVENT) is a partial support mode that employs pressure-controlled, time-cycled ventilation set at two levels of continuous positive airway pressure (CPAP) with unrestricted spontaneous breathing. It may be used in any phase of the mechanical ventilatory cycle. Biphasic positive airway pressure is able to modulate the inspiratory effort by modifying the frequency of controlled breaths. Nevertheless, the optimal amount of inspiratory effort to improve respiratory function while minimizing ventilator-associated lung injury (VALI) during partial ventilatory assistance has not been determined. Moreover, it is unclear whether the effects of partial ventilatory support depend on ARDS etiology. Theoretically, pulmonary ARDS (ARDS_p_) involves higher degrees of lung tissue consolidation, whereas extrapulmonary ARDS (ARDS_exp_) is associated mainly with alveolar collapse, which can potentially be overcome by increased inspiratory effort [9].

In the present study, we investigated the impact of inspiratory effort during biphasic positive airway pressure on lung morphology and function, markers of inflammation, fibrosis, apoptosis, endothelial and epithelial cell damage, and diaphragmatic injury in experimental pulmonary and extrapulmonary acute lung injury (ALI_p_ and ALI_exp_, respectively). We hypothesized that biphasic positive airway pressure would (1) improve lung function and reduce VALI compared to pressure-controlled ventilation (PCV), (2) modulate lung injury according to the frequency of time-cycled control breaths and inspiratory effort and (3) have etiology-dependent effects on breathing patterns, lung mechanics, histology and biochemical response.

## Materials and methods

This study was approved by the Research Ethics Committee of the Federal University of Rio de Janeiro Health Sciences Center. All animals received humane care in compliance with the Principles of Laboratory Animal Care formulated by the National Society for Medical Research and the Guide for the Care and Use of Laboratory Animals prepared by the U.S. National Academy of Sciences.

### Animal preparation and experimental protocol

Sixty adult male Wistar rats (250 to 300 g) were kept under specific pathogen-free conditions in an animal care facility at the Federal University of Rio de Janeiro. Mild ALI was induced in all animals by *Escherichia coli* O55:B5 lipopolysaccharide (LPS). Because ALI etiology might entail different effects of partial ventilatory support, both ALI_p_ and ALI_exp_ were induced by intratracheal or intraperitoneal injection of *E. coli* LPS (200 μg for ALI_p_ and 1,000 μg for ALI_exp_ suspended in saline solution with total volumes equal to 100 μl and 1,000 μl, respectively) [10]. The animals were randomly allocated to the ALI_p_ or ALI_exp_ group.

Twenty-four hours after ALI induction, the rats were sedated (10 mg/kg diazepam intraperitoneally), anesthetized (100 mg/kg ketamine and 10 mg/kg xylazine intraperitoneally) and tracheotomized. Twelve of the sixty rats (*n* = 6 per ALI etiology) were used for electron microscopy and molecular biology analysis and were not mechanically ventilated (nonventilated, NV).

**Figure 1:**
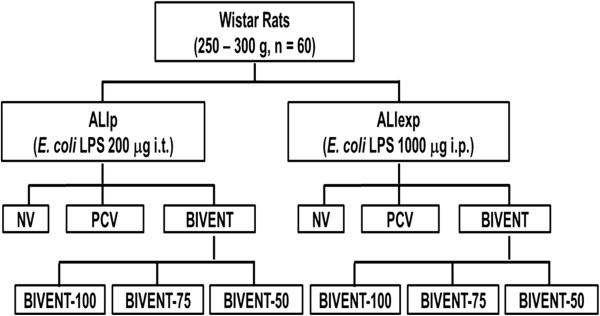
**Schematic flowchart of study design.** ALI_exp_: extrapulmonary acute respiratory distress syndrome; ALI_p_: pulmonary acute respiratory distress syndrome; BIVENT: biphasic positive airway pressure at different rates of time-cycled controlled breaths (100, 75 and 50 breaths/min); *E. coli* LPS: *Escherichia coli* O55:B5 lipopolysaccharide; i.p.: intraperitoneal; i.t.: intratracheal; NV: nonventilated; PCV: pressure-controlled ventilation.

**Figure 2:**
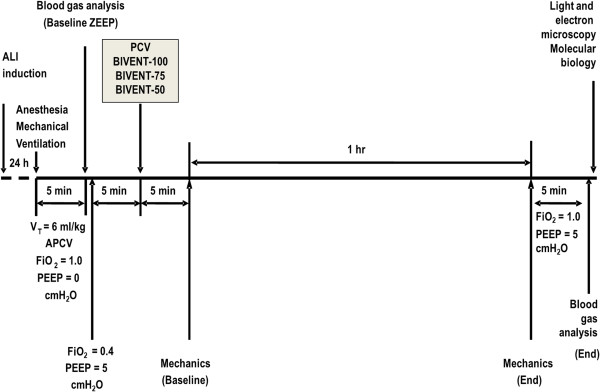
**Timeline of the procedure.** ALI: acute lung injury; APCV: assisted pressure-controlled ventilation; BIVENT: biphasic positive airway pressure; FiO_2_: fraction of inspired oxygen; PCV: pressure-controlled ventilation; PEEP: positive end-expiratory pressure; V_t_: tidal volume; ZEEP: zero end-expiratory pressure.

A polyethylene catheter (PE-10) was introduced into the carotid artery for blood sampling and monitoring of mean arterial pressure (MAP). Electrocardiograms, MAP and rectal temperature were continuously recorded (Networked Multi-Parameter Veterinary Monitor LifeWindow 6000 V; Digicare Animal Health, Boynton Beach, FL, USA). The tail vein was punctured for continuous infusion of Ringer’s lactate solution (10 ml/kg/h). Gelafundin (B. Braun, Melsungen, Germany) was administered (in 0.5-ml increments) to keep MAP above 70 mmHg. Animals were mechanically ventilated (SERVO-i; MAQUET, Solna, Sweden) in volume-controlled mode at the following settings: V_t_ = 6 ml/kg, respiratory rate (RR) = 100 breaths/min, fraction of inspired oxygen (FiO_2_) = 1.0, inspiratory-to-expiratory ratio (I:E) = 1:2 and zero end-expiratory pressure (ZEEP) during five minutes. Arterial blood (300 μl) was drawn into a heparinized syringe for measurement of arterial oxygen partial pressure (PaO_2_), partial pressure of arterial carbon dioxide (PaCO_2_) and arterial pH (pH_a_) (i-STAT; Abbott Laboratories, Abbott Park, IL, USA) (baseline ZEEP). Following this step, the animals were randomly assigned to one of the following mechanical ventilation groups: (1) PCV mode with V_t_ = 6 ml/kg, RR = 100 breaths/min, PEEP = 5 cmH_2_O and I:E = 1:2, during which animals were paralyzed with pancuronium bromide (2 mg/kg intravenously); or (2) BIVENT with three different rates of time-cycled control breaths (100, 75 and 50 breaths/min). In both groups, BIVENT was set at two levels of CPAP (P_high_ = 10 cmH_2_O and P_low_ = 5 cmH_2_O). Inspiratory time was kept constant (T_high_ = 0.3 seconds). Both ventilator strategies were maintained with FiO_2_ = 0.4 for one hour. FiO_2_ was then set at 1.0 for five minutes, and arterial blood gases were analyzed (End). Lungs were extracted for histological and molecular biological analysis. Schematic flowcharts of the study design and the timeline representation of the procedures are shown in Figures 1 and 2, respectively.

### Data acquisition and processing

**Figure 3:**
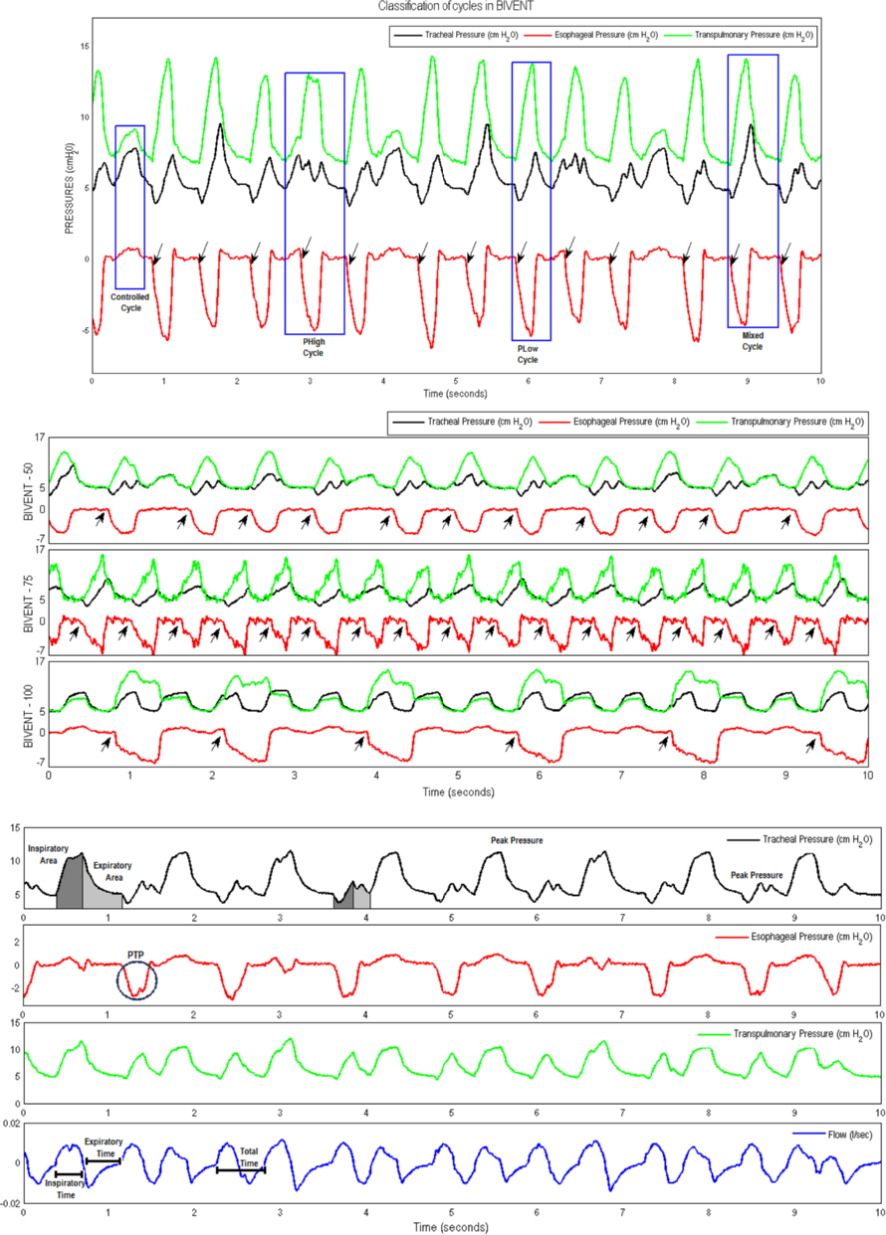
**Original tracheal, esophageal and transpulmonary pressure and airflow tracings.** Top panel: Original tracings of tracheal (black line), esophageal (red line) and transpulmonary (green line) pressure. Note the two levels of continuous positive airway pressure (P_high_ = 10 cmH_2_O and P_low_ = 5 cmH_2_O) in controlled and mixed cycles (blue boxes). Black arrows in top and middle panels indicate beginning of inspiration. Middle panel: Original tracings of tracheal (black line), esophageal (red line) and transpulmonary (green line) pressure at different rates of time-cycled controlled breaths: 100, 75 and 50 breaths/min. Bottom panel: Original tracings of esophageal and transpulmonary pressure and airflow. Blue circles: PTP area. BIVENT: biphasic positive airway pressure; P_high_: high level of continuous positive airway pressure; P_low_: low level of continuous positive airway pressure; PTP: pressure–time product per breath.

After one hour of mechanical ventilation, all variables were recorded for ten minutes. Airway pressure (P_aw_) was measured with a differential pressure transducer (UT-PDP-300; SCIREQ, Montreal, QC, Canada). Changes in esophageal pressure (P_es_), which reflect chest wall pressure, were measured with a 30-cm-long, water-filled catheter (PE205) with side holes at the tip connected to a differential pressure transducer (UT-PL-400; SCIREQ). The catheter was passed into the stomach and then slowly withdrawn back into the esophagus. Its proper positioning was assessed using the occlusion test [11]. V_t_ was calculated by digital integration of the flow signal. P_L_ was calculated during inspiration and expiration as the difference between tracheal and esophageal pressure. Mean airway pressure (P_mean,aw_) and peak airway pressure (P_peak,aw_) were calculated. The total RR was calculated from the P_es_ swings as the frequency per minute of each type of breathing cycle. The pressure–time product (PTP) per breath was calculated as the integral of ∆P_es_ over time. ∆P_es_ was measured from the beginning of inspiration during each type of breathing cycle, independent of the CPAP level. Total ventilation (V′_e,tot_) and PTP per minute were calculated by multiplying the V_t_ and PTP by the corresponding frequency at one minute for each breathing cycle, respectively. RR, V_t_, V′_e_, and PTP were calculated for three different types of breathing cycles as follows: (1) fully controlled cycles (C) as time-cycled breaths that were not accompanied by negative P_es_ swings; (2) spontaneous breath cycles at high and low CPAP levels (P_high_ and P_low_, respectively) as negative P_es_ swings not followed by ventilator cycling; and (3) mixed respiratory cycles (M) as negative P_es_ swings with simultaneous ventilator inspiratory cycling (Figure 3).

Airflow, tracheal and esophageal pressure were continuously recorded throughout the experiments with a computer running software written in LabVIEW (National Instruments, Austin, TX, USA). All signals were filtered (200 Hz), amplified by a four-channel conditioner (SC-24; SCIREQ) and sampled at 200 Hz with a 12-bit analog-to-digital converter (National Instruments). All mechanical data were computed offline by a routine written in MATLAB software (version R2007a; The MathWorks Inc, Natick, MA, USA).

### Histology

#### Light microscopy

A laparotomy was performed immediately after blood sampling at the end of the experiments. Heparin (1,000 IU) was injected into the tail vein. Sodium thiopental (25 mg/ml) was injected to increase the level of anesthesia, and the trachea was then clamped at end expiration (PEEP = 5 cmH_2_O). The abdominal aorta and vena cava were sectioned, yielding a massive hemorrhage that quickly killed the animals. Lungs were removed *en bloc* with end expiratory pressure of 5 cmH_2_O in all groups to avoid distortion of lung morphometry. The left lung was frozen in liquid nitrogen and immersed in Carnoy’s solution. Lung morphometric analysis was performed using an integrating eyepiece with a coherent system consisting of a grid with 100 points and 50 lines of known length coupled to a conventional light microscope (Olympus BX51; Olympus Latin America, Rio de Janeiro, Brazil). The volume fractions of the lung occupied by collapsed alveoli, normal pulmonary areas or hyperinflated structures (alveolar ducts, alveolar sacs or alveoli; maximal chord length in air greater than 120 μm) were determined by the point-counting technique at a magnification of ×200 across ten random, noncoincident microscopic fields [12].

#### Transmission electron microscopy

Three slices measuring 2 × 2 × 2 mm each were cut from three different segments of the right lung and diaphragm. They were then fixed (2.5% glutaraldehyde and phosphate buffer 0.1 M, pH 7.4) for electron microscopy analysis (JEOL 1010 Transmission Electron Microscope; JEOL, Tokyo, Japan). For each electron microscopy image (20 per animal), an injury score was calculated. The following parameters were analyzed concerning lung parenchyma: damage to alveolar capillary membrane, type II epithelial cell lesion and endothelial cell damage [10]. The following aspects were assessed on the basis of electron microscopy of the diaphragm muscle: (1) myofibrillar abnormalities, defined as disruption of myofibrillar bundles or disorganized myofibrillar pattern with edema of the Z disks, a filamentous network of proteins forming a disklike structure for the attachment of actin myofilaments (The Z disks provide structural linkage for the transmission of tension and contractile forces along the muscle fiber and play a role in sensing muscle activity and signal transduction); (2) mitochondrial injury with abnormal, swollen mitochondria and abnormal cristae; and (3) miscellaneous, which included lipid droplets, vacuoles, intermyofibril space and nuclei. The pathological findings were graded according to a five-point, semiquantitative, severity-based scoring system expressed as percentage of examined tissue: 0 = normal lung parenchyma or diaphragm, 1 = changes in 1% to 25%, 2 = changes in 26% to 50%, 3 = changes in 51% to 75% and 4 = changes in 76% to 100%. The pathologist or technician working on the electron microscopy images was blinded to the nature of the study.

### Biological markers of apoptosis, fibrogenesis and lung epithelial and endothelial cell damage

Quantitative real-time reverse transcription polymerase chain reactions were performed to measure biological markers associated with apoptosis (procaspase 3); fibrogenesis (type III procollagen, PC_III_); damage inflicted on alveolar type I (receptor for advanced glycation end product, RAGE) and alveolar type II epithelial cells (surfactant protein B); and endothelium (vascular cellular adhesion molecule 1 (VCAM-1) and intercellular adhesion molecule 1 (ICAM-1) [13] (Table 1). Central slices of right lung tissue were cut, collected in cryotubes, quick-frozen by immersion in liquid nitrogen and stored at -80°C. Total RNA was extracted from frozen tissue using the SV Total RNA Isolation System (Promega, Madison, WI, USA), following the manufacturer’s recommendations. RNA concentration was measured by spectrophotometry in a NanoDrop ND-1000 System (NanoDrop Products, Wilmington, DE, USA). First-strand cDNA was synthesized from total RNA using the GoTaq 2-Step RT-qPCR System (Promega). Relative mRNA levels were measured by SYBR Green–based detection using the ABI 7500 Real-Time PCR System (Applied Biosystems, Foster City, CA, USA). Samples were measured in triplicate. For each sample, the expression of each gene was normalized to housekeeping gene expression (acidic ribosomal phosphoprotein P0, 36B4) using the 2^-ΔΔCt^ method, where ΔCt = Ct (reference gene) - Ct (target gene).

**Table 1:** **Target gene primers**^**a**^

**Target gene**	**Sense**	**Antisense**
*RAGE*	5′-TGAACTCACAGCCAATGTCC-3′	5′-ACAACTGTCCCTTTGCCATC-3′
*SP-B*	5′-CTGTGCCAAGAGTGTGAGGA-3′	5′-CAAGCAGCTTCAAGGGTAGG-3′
*VCAM-1*	5′-TGCACGGTCCCTAATGTGTA-3′	5′-TGCCAATTTCCTCCCTTAAA-3′
*ICAM-1*	5′-CTTCCGACTAGGGTCCTGAA-3′	5′-CTTCAGAGGCAGGAAACAGG-3′
*PCIII*	5′-ACCTGGACCACAAGGACAC-3′	5′-TGGACCCATTTCACCTTTC-3′
*Procaspase 3*	5′-GGCCGACTTCCTGTATGC-3′	5′-GCGCAAAGTGACTGGATG- 3′
*36B4*	5′-AATCCTGAGCGATGTGCAG-3′	5′-GCTGCCATTGTCAAACAC-3′

^a^RAGE: receptor for advanced glycation end products, SP-B: surfactant protein B, VCAM-1: vascular cellular adhesion molecule 1, ICAM-1: intercellular adhesion molecule 1. PCIII: type III procollagen.

### Statistical analysis

Data were tested for normal distribution (Kolmogorov-Smirnov test with Lilliefors correction) and homogeneity of variances (Levene median test). If both conditions were met, the effects of different ventilatory strategies on ALI groups were analyzed by means of one-way analysis of variance (ANOVA) followed by Tukey’s test. Otherwise, one-way ANOVA on ranks followed by Dunn’s *post hoc* test was employed. The significance level was always set at 5%. Parametric data were expressed as means ± SD, and nonparametric data were expressed as medians (IQR). All tests were performed using SigmaStat 3.1 software (Jandel Corp, San Raphael, CA, USA).

## Results

**Figure 4:**
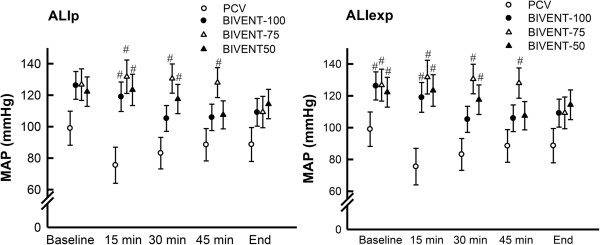
**Mean arterial pressure of animals with pulmonary and extrapulmonary acute respiratory distress syndrome.** ALI_exp_: extrapulmonary acute lung injury; ALI_p_: pulmonary acute lung injury; BIVENT: biphasic positive airway pressure; MAP: mean arterial pressure; PCV: pressure-controlled ventilation. Values are means ± SD of six rats at each time point. ^#^Significantly different from PCV (*P* < 0.05).

Mean arterial pressure was higher than 70 mmHg throughout the experiments in both ALI groups, but was significantly lower during PCV than during BIVENT (Figure 4). In the ALI_exp_ group, animals required additional fluid administration to keep MAP higher than 70 mmHg (*P* < 0.05).

At baseline, the overall respiratory mechanics parameters were comparable among groups, except for PTP, which was lower for BIVENT-100 in the ALI_p_ group (Table 2). At the end of the experiment, minutes on ventilation, mean V_t_ and peak airway pressure were comparable in the PCV and BIVENT groups, regardless of ALI etiology. The RR of spontaneous breaths, minutes on ventilation, PTP/min and P_0.1_ during P_low_ were significantly increased for BIVENT-50 compared to BIVENT-75 and BIVENT-100. Inspiratory pressure and mean airway pressure were similar in all assisted ventilation modes (Table 3). However, with regard to spontaneous breaths during P_low_, P_0.1_ was higher for BIVENT-50 than for BIVENT-75 and BIVENT-100. PaO_2_, PaCO_2_ and pH_a_ at baseline ZEEP and after one hour of ventilation (End) did not differ among ventilation strategies and ALI groups (Table 4).

**Table 2:** **Baseline mechanical data**^**a**^

**Parameters**		**ALI**_**p**_				**ALI**_**exp**_			
		**PCV**	**BIVENT-50**	**BIVENT-75**	**BIVENT-100**	**PCV**	**BIVENT-50**	**BIVENT-75**	**BIVENT-100**
RR (breaths/min)	C	99 (99 to 99)	42 (25 to 49)	55 (0 to 72)	96 (65 to 97)	99 (98 to 99)	22 (0 to 43)	59 (32 to 73)	69 (48 to 96)
	M	–	0 (0 to 13)	0 (0 to 73)	0 (0 to 0)	–	11.5 (0 to 48.0)	0 (0 to 30.0)	0 (0 to 0)
	P_high_	–	0 (0 to 4)	0 (0 to 16)	0 (0 to 30.0)	–	1.5 (0 to 7)	0 (0 to 0)	11 (0-32)
	P_low_	–	33 (0 to 49)	–	–	–	0 (0 to 47)	0 (0 to 55.0)	–
	Total	99 (99 to 99)	80 (51 to 92)	73 (73 to 73)	97 (96 to 97)	99 (98 to 99)	50 (49 to 92)	75 (73 to 111)	96 (96 to 97)
V_t_ (ml)	C	1.7 (1.6 to 1.8)	1.4 (0.92 to 1.70)	0.7 (0 to 1.4)	1.5 (1.4 to 1.6)	1.7 (1.5 to 1.8)	1.17 (0 to 1.3)	0.7 (0.4 to 1.4)	1.3 (0.8 to 1.5)
	M	–	0 (0 to 1.9)	0 (0 to 1.8)	0 (0 to 0)	–	0.8 (0 to 1.8)	0 (0 to 1.7)	0 (0 to 0)
	P_high_	–	0 (0 to 1.7)	0 (0 to 1.2)	0 (0 to 1.6)	–	0.7 (0 to 1.5)	0 (0 to 0)	0.5 (0 to 1.7)
	P_low_	–	1.1 (0 to 1.5)	–	–	–	0 (0 to 1.7)	0 (0 to 2.2)	–
	Total	1.7 (1.6 to 1.8)	1.5 (1.5 to 1.6)	1.5 (1.0 to 1.8)	1.6 (1.4 to 1.6)	1.7 (1.5 to 1.8)	1.6 (1.4 to 1.6)	1.5 (1.3 to 1.7)	1.3 (1.2 to 1.6)
V′_e_ (ml/min)	C	168 (159 to 177)	51 (28 to 83)	38 (0 to 101)	141 (95 to 156)	161 (130 to 182)	33 (0 to 54)	32 (23 to 102)	82 (43 to 121)
	M	–	0 (0 to 24.8)	0 (0 to 139)	0 (0 to 0)	–	20 (0 to 73)	0 (0 to 52)	0 (0 to 0)
	P_high_	–	0 (0 to 6.8)	0 (0 to 22)	0 (0 to 54)	–	2 (0 to 9)	0 (0 to 0)	19 (0 to 63)
	P_low_	–	40 (0 to 67)	0 (0 to 0)	0 (0 to 0)	–	0 (0 to 85)	0 (0 to 136)	–
	Total	168 (159 to 177)	107 (83 to 145)	106 (72 to 139)	150 (144 to 156)	161 (130 to 182)	85 (71 to 149)	141 (102 to 160)	121 (111 to 151)
P_peak,aw_ (cmH_2_O)	C	15.6 (14.2 to 18.8)	11.8 (10.4 to 13.0)	11.4 (11 to 12.1)	15.5 (13.7 to 17.9)	13.5 (13 to 14.7)	10.8 (10.5 to 12.4)	9.8 (9.3 to 12.5)	13.3 (9.5 to 13.9)
	M	–	11.1 (9.8 to 12.4)	11.4 (11.0 to 11.9)	15.2 (15.2 to 15.2)	–	12.7 (11.4 to 12.9)	10.5 (9.1 to 11.9)	9.2 (9.2 to 9.2)
P_mean,aw_ (cmH_2_O)	C	9.2 (8.3 to 10.0)	7.6 (6.7 to 8.4)	7.5 (7.4 to 7.7)	10.5 (9.2 to 11.0)	8.7 (8.5 to 9.5)	7.6 (7.2 to 7.8)	8.0 (7.6 to 8.1)	9.1 (7.3 to 9.4)
	M	–	6.6 (6.1 to 7.0)	7.1 (6.7 to 7.5)	9.1 (9.1 to 9.1)	–	6.8 (6.6 to 7.0)	6.8 (6.4 to 7.2)	6.8 (6.8 to 6.8)
T_i_/T_tot_	C	0.35 (0.35 to 0.36)	0.36 (0.27 to 0.44)	0.36 (0 to 0.39)	0.50 (0.50 to 0.51)	0.36 (0.35 to 0.37)	0.27 (0 to 0.45)	0.41 (0.38 to 0.51)	0.51 (0.49 to 0.51)
PEEP		5.0	5.0	5.0	5.0	5.0	5.0	5.0	5.0
PTP (cmH_2_O/ml)	M	–	0 (0 to 38.7)	0 (0 to 143.2)	0 (0 to 0)	–	0 (0 to 8.7)	0 (0 to 54.3)	0 (0 to 0)
	P_high_	–	0 (0 to 3.2)	0 (0 to 37.7)	0 (0 to 38.2)	–	0 (0 to 4.0)	0 (0 to 0)	22.0 (0 to 68.8)
	P_low_	–	64.7 (0 to 134.5)	–	–	–	0 (0 to 0)	0 (0 to 168.2)	–
	Total	–	86.8 (76.4 to 137.7)	67.4 (0 to 143.2)	0 (0 to 38.2)^b,c^	–	40.6 (9.3 to 57.6)	90.5 (0 to 168.2)	56.4 (0 to 113.0)
P_0.1_ (cmH_2_O)	M	–	0 (0 to 0.3)	0 (0 to 0.5)	0 (0 to 0)	–	0.1 (0 to 0.9)	0 (0 to 1.4)	0 (0 to 0)
	P_high_	–	0 (0 to 1.2)	0 (0 to 0.9)	0 (0 to 0.6)	–	0.2 (0 to 0.7)	0 (0 to 0)	0.4 (0 to 2.2)
	P_low_	–	0.5 (0 to 0.7)	–	–	–	0 (0 to 0.9)	0 (0 to 2.9)	–

^a^ALI_exp_: extrapulmonary acute lung injury; ALI_p_: pulmonary acute lung injury; BIVENT: biphasic positive airway pressure at different rates of time-cycled controlled breaths: 100, 75 and 50 breaths/min; C = fully controlled breaths; M = mixed, assisted breaths; P_0.1_: driving pressure; PCV: pressure-controlled ventilation; PEEP: positive end-expiratory pressure; P_high_ = spontaneous breaths at high continuous positive airway pressure; P_low_ = spontaneous breaths at low continuous positive airway pressure; P_mean,aw_: mean airway pressure; P_peak,aw_: peak airway pressure; PTP: pressure–time product per breath; RR: respiratory rate; V′_e_: minutes on ventilation; V_t_: tidal volume; T_i_/T_tot_: inspiratory time divided by total respiratory cycle time. Values are medians and IQRs of six rats per group. ^b^BIVENT-100, ^c^BIVENT-75 (*P* < 0.05).

**Table 3:** **Mechanical data at end**^**a**^

		**ALI**_**p**_				**ALI**_**exp**_			
**Parameters**		**PCV**	**BIVENT-100**	**BIVENT-75**	**BIVENT-50**	**PCV**	**BIVENT-100**	**BIVENT-75**	**BIVENT-50**
RR (breaths/min)	C	99 (98 to 99)	23 (0 to 48)^b^	21 (0 to 45)^b^	20 (0 to 22)^b^	99 (79 to 99)	63 (30 to 64)	0 (0 to 0)^b^	8 (0 to 17)^b^
	M	–	13 (0 to 29)	0 (0 to 31)	8 (4 to 14)^c,d^	–	0 (0 to 0)	54 (33 to 74)	33 (8 to 49)
	P_high_	–	45 (18 to 48)	26 (0 to 72)	22 (11 to 22)	–	32 (7 to 34)	18 (0 to 39)	0 (0 to 16)
	P_low_	–	–	–	44 (41 to 44)^c,d^	–	0 (0 to 0)	0 (0 to 0)	21 (0 to 49)
	Total	99 (98 to 99)	104 (96 to 129)	83 (74 to 105)^b^	72 (49 to 98)	99 (79 to 99)	97 (96 to 110)	73 (72 to 76)	92 (91 to 97)
V_t_ (ml)	C	1.8 (1.7 to 1.9)	0.7 (0 to 1.7)	0.4 (0 to 1.2)^b^	0.6 (0 to 1.4)	1.7 (1.7 to 1.8)	0.7 (0.3 to 1.2)^b^	0 (0 to 0)^b^	0.3 (0 to 0.6)^b^
	M	–	0.4 (0 to 2.0)	0 (0 to 1.4)	2.4 (2.2 to 3.2)^c,d^	–	0 (0 to 0)	2.3 (1.6 to 2.6)^d^	1.7 (1.4 to 2.2)
	P_high_	–	2.0 (1.8 to 2.4)	1.7 (0 to 1.9)	2.2 (1.9 to 3.2)	–	2.2 (1.7 to 2.4)	1.17 (0.0 to 2.6)	0 (0 to 1.9)
	P_low_	–	–	–	1.9 (1.5 to 2.1)^c,d^	–	0 (0 to 0)	0 (0 to 0)	0.9 (0 to 1.9)
	Total	1.8 (1.7 to 1.9)	1.4 (1.3 to 1.8)	2.3 (1.6 to 2.6)	1.6 (1.4 to 2.0)	1.7 (1.7 to 1.8)	1.5 (1.3 to 1.7)	1.7 (1.4 to 2.2)	1.8 (1.6 to 2.3)
V′_e_ (ml/min)	C	176 (164 to 185)	24 (0 to 38)^b^	15 (0 to 66)^b^	12 (0 to 41)^b^	168 (139 to 171)	34 (20 to 75)	0 (0 to 0)^b^	4 (0 to 10)^b^
	M	–	11 (0 to 58)	0 (0 to 42)	19 (10 to 49)^c^	–	0 (0 to 0)	107 (85 to 178)	54 (22 to 78)
	P_high_	–	98 (37 to 124)	42 (0 to 142)	48 (21 to 67)	–	78 (15 to 80)	42 (0 to 105)	0 (0 to 31)
	P_low_	–	0 (0 to 0)	0 (0 to 0)	79 (66 to 90)^c,d^	–	0 (0 to 0)	0 (0 to 0)	43 (0 to 91)
	Total	176 (164 to 185)	168 (152 to 194)	125 (101 to 158)	156 (147 to 214)	168 (139 to 171)	154 (147 to 165)	190 (118 to 263)	115 (78 to 171)
P_peak,aw_ (cmH_2_O)	C	13.6 (13.2 to 15.1)	10.6 (8.8 to 13.5)	11.3 (10.9 to 13.1)	11.1 (9.6 to 11.6)	13.5 (13.1 to 13.5)	9.3 (9.2 to 11.8)	13.7 (13.7 to 13.7)	9.3 (8.2 to 10.5)
	M	–	12.9 (10.6 to 14.3)	12.9 (12.1 to 13.6)	11.5 (10.8 to 12.2)	–	10.7 (10.7 to 10.7)	9.3 (8.3 to 12.0)	11.0 (10.2 to 12.6)
P_mean,aw_ (cmH_2_O)	C	9.0 (8.8 to 9.6)	7.8 (7.1 to 9.5)	7.5 (7.4 to 8.2)	8.3 (7.7 to 8.4)	8.8 (8.7 to 9.0)	8.2 (7.2 to 10.2)	8.6 (8.6 to 8.6)	8.0 (7.0 to 13.4)
	M	–	8.6 (7.0 to 9.4)	7.9 (7.4 to 8.3)	7.1 (6.4 to 7.4)	–	7.3 (7.3 to 7.3)	6.0 (5.5 to 7.2)	6.7 (6.5 to 6.8)
T_i_/T_tot_ (s)	C	0.36 (0.35 to 0.37)	0.48 (0 to 0.58)	0.18 (0 to 0.38)	0.46 (0 to 0.47)	0.35 (0.35 to 0.37)	0.50 (0.47 to 0.51)	0 (0 to 0)^d^	0.24 (0 to 0.58)
PEEP (cmH_2_O)		5.0	5.0	5.0	5.0	5.0	5.0	5.0	5.0
PTP (cmH_2_O/ml)	M	–	49.9 (0 to 138.3)	0 (0 to 8.3)	37.0 (74.2 to 14.1)^c^	–	0 (0 to 0)	42.3 (30.4 to 103.4)^d^	12.1 (0 to 17.0)
	P_high_	–	152.7 (19.4 to 321.6)	46.2 (0 to 128.6)	45.4 (5.2 to 66.2)^d^	–	50.3 (16.6 to 101.2)	11.3 (0 to 34.1)	0 (0 to 14.9)^d^
	P_low_	–	0 (0 to 0)	0 (0 to 0)	174.4 (89.0 to 251.6)^c,d^	–	0 (0 to 0)	0 (0 to 0)	25.5 (0 to 99.3)^c,d^
	Total	–	254.7 (171.1 to 372.2)	96.0 (29.1 to 217.8)	274.0 (152.8 to 323.8)	–	78.3 (45.3 to 123.9)	78.3 (44.0 to 122.5)	60.6 (10.5 to 99.3)^c,d^
P_0.1_ (cmH_2_O)	M	–	0.2 (0 to 0.9)	0 (0 to 0.1)	1.1 (0.9 to 1.3)^c,d^	–	0 (0 to 0)	1.0 (0.6 to 1.3)^d^	0.2 (0 to 0.8)
	P_high_	–	0.5 (0.2 to 1.5)	0.5 (0 to 1.1)	0.7 (0.3 to 1.0)	–	2.2 (1.3 to 2.8)	0.3 (0 to 1.0)	0 (0 to 0.9)
	P_low_	–	0 (0 to 0)	0 (0 to 0)	1.3 (0.6 to 1.5)^c,d^	–	0 (0 to 0)	0 (0 to 0)	0.5 (0 to 2.0)^c,d^

^a^ALI_exp_: extrapulmonary acute lung injury; ALI_p_: pulmonary acute lung injury; BIVENT: biphasic positive airway pressure at different rates of time-cycled controlled breaths: 100, 75 and 50 breaths/min; C = fully controlled breaths; M = mixed, assisted breaths; P_0.1_: driving pressure; PCV: pressure-controlled ventilation; PEEP: positive end-expiratory pressure; P_high_ = spontaneous breaths at high continuous positive airway pressure; P_low_ = spontaneous breaths at low continuous positive airway pressure; P_mean,aw_: mean airway pressure; P_peak,aw_: peak airway pressure; PTP: pressure–time product per breath; RR: respiratory rate; V′_e_: minutes on ventilation; V_t_: tidal volume; T_i_/T_tot_: inspiratory time divided by total respiratory cycle time. Values are medians and IQRs of six rats per group. ^b^PCV, ^c^BIVENT-75, ^d^BIVENT-100 (*P* < 0.05).

**Table 4:** **Arterial blood gases at baseline zero end-expiratory pressure and end**^**a**^

	**ALI**_**p**_				**ALI**_**exp**_			
**Arterial blood gases**	**PCV**	**BIVENT-100**	**BIVENT-75**	**BIVENT-50**	**PCV**	**BIVENT-100**	**BIVENT-75**	**BIVENT-50**
Baseline ZEEP								
PaO_2_ (mmHg)	177.5 ± 42.3	153.0 ± 39.2	162.0 ± 46.7	227.0 ± 56.6	149.7 ± 15.8	169.5 ± 31.8	181.8 ± 28.4	174.5 ± 26.5
PaCO_2_ (mmHg)	41.7 ± 5.1	42.0 ± 2.9	47.7 ± 2.3	42.2 ± 1.9	50.2 ± 6.6	43.4 ± 2.7	44.2 ± 3.2	42.1 ± 2.9
pH_a_	7.27 ± 0.03	7.31 ± 0.02	7.29 ± 0.02	7.30 ± 0.03	7.20 ± 0.03	7.27 ± 0.02	7.24 ± 0.04	7.29 ± 0.04
End								
PaO_2_ (mmHg)	457.7 ± 35.6	458.8 ± 16.9	410.5 ± 53.1	456.2 ± 35.3	393.7 ± 30.4	350.8 ± 20.9	395.8 ± 23.7	381.3 ± 7.6
PaCO_2_ (mmHg)	37.8 ± 5.1	43.1 ± 2.5	44.2 ± 4.3	41.8 ± 1.0	44.1 ± 1.6	43.2 ± 1.5	47.5 ± 3.9	48.4 ± 2.0
pH_a_	7.27 ± 0.03	7.31 ± 0.03	7.24 ± 0.05	7.29 ± 0.02	7.21 ± 0.02	7.20 ± 0.03	7.26 ± 0.04	7.27 ± 0.03

^a^ALI_exp_: extrapulmonary acute lung injury; ALI_p_: pulmonary acute lung injury; BIVENT: biphasic positive airway pressure at different rates of time-cycled controlled breaths: 100, 75 and 50 breaths/min; PaO_2_: partial pressure of arterial oxygen; PaCO_2_: partial pressure of arterial carbon dioxide; PCV: pressure-controlled ventilation;, pH_a_: arterial pH; ZEEP: zero end-expiratory pressure. PaO_2_, PaCO_2_ and pH_a_ were measured at baseline ZEEP and after one hour of mechanical ventilation at a fraction of inspired oxygen = 1.0 in animals with ALI_p_ and ALI_exp_. Values are means ± SEM of six rats.

**Figure 5:**
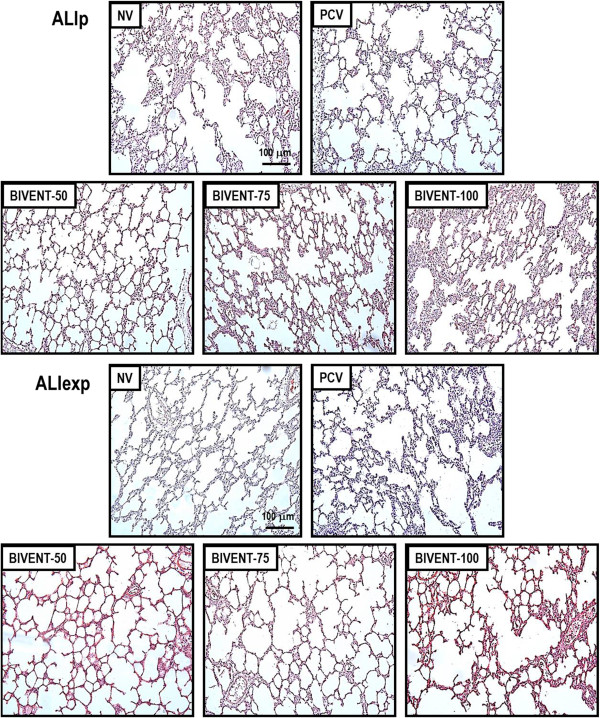
**Photomicrographs of lung parenchyma stained with hematoxylin and eosin.** Photomicrographs are representative of data obtained from lung sections of six animals (original magnification, ×200). ALI_exp_: extrapulmonary acute lung injury; ALI_p_: pulmonary acute lung injury; BIVENT: biphasic positive airway pressure at different rates of time-cycled controlled breaths: 100, 75 and 50 breaths/min; NV: nonventilated; PCV: pressure-controlled ventilation.

In ALI_p_, alveolar collapse was higher at BIVENT-100 compared to PCV and lower at BIVENT-50 than at BIVENT-100 and BIVENT-75. In ALI_exp_, alveolar collapse was lower at BIVENT-50 than the other ventilatory strategies (Table 5 and Figure 5).

**Table 5:** **Lung morphometry**^**a**^

	**ALI**_**p**_					**ALI**_**exp**_				
**Morphometry**	**NV**	**PCV**	**BIVENT-100**	**BIVENT-75**	**BIVENT-50**	**NV**	**PCV**	**BIVENT-100**	**BIVENT-75**	**BIVENT-50**
Normal (%)	64.1 ± 15.8	72.1 ± 5.5	52.2 ± 10.3^b^	65.2 ± 6.1	84.6 ± 2.6^c,d,e^	72.4 ± 4.0	69.3 ± 3.9	67.5 ± 6.2	72.0 ± 9.0	88.1 ± 1.9^b,c,d,e^
Collapsed (%)	33.7 ± 13.3	26.6 ± 6.3	45.5 ± 10.8^b^	32.9 ± 5.0	15.1 ± 2.2^c,d,e^	24.0 ± 5.4	27.7 ± 5.9	30.8 ± 6.7	26.7 ± 9.6	11.9 ± 1.9^b,c,d,e^
Hyperinflated (%)	2.1 ± 6.4	1.3 ± 2.5	2.2 ± 1.2	1.9 ± 1.4	0.3 ± 0.7	3.0 ± 3.0	3.0 ± 2.0	1.7 ± 1.3	1.3 ± 2.8	0 ± 0

^a^ALI_exp_: extrapulmonary acute lung injury; ALI_p_: pulmonary acute lung injury; BIVENT: biphasic positive airway pressure at different rates of time-cycled controlled breaths: 100, 75 and 50 breaths/min; NV: nonventilated; PCV: pressure-controlled ventilation. Data are volume fractions of the lung occupied by normal pulmonary areas, collapsed alveoli and hyperinflated structures in animals with ALI_p_ and ALI_exp_. . All values were computed in ten random, noncoincident fields per rat. Values are means ± SD of six rats in each group. ^b^PCV, ^c^NV, ^d^BIVENT-100, ^e^BIVENT-75 (*P* < 0.05).

**Figure 6:**
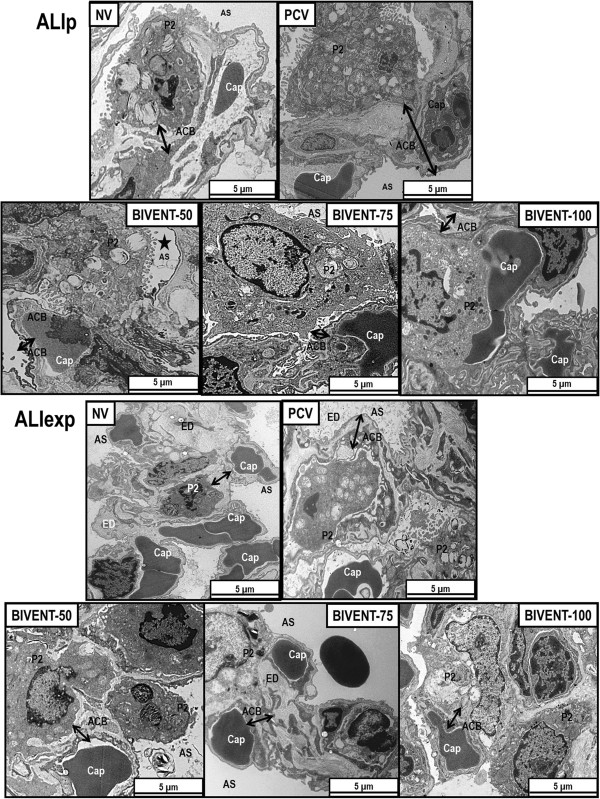
**Electron microscopy of lung parenchyma.** Photomicrographs are representative of data obtained from lung sections of five animals per group. Black arrows: alveolar capillary basement (ACB) membrane. Note that endothelial cells as well as alveolar types I and II epithelial cells were injured. ALI_exp_: extrapulmonary acute lung injury; ALI_p_: pulmonary acute lung injury; AS = intra-alveolar space; BIVENT: biphasic positive airway pressure at different rates of time-cycled controlled breaths: 100, 75 and 50 breaths/min; Cap = capillary; E = erythrocyte; ED = edema; NV: nonventilated; P2 = type II epithelial cell; PCV: pressure-controlled ventilation.

**Figure 7:**
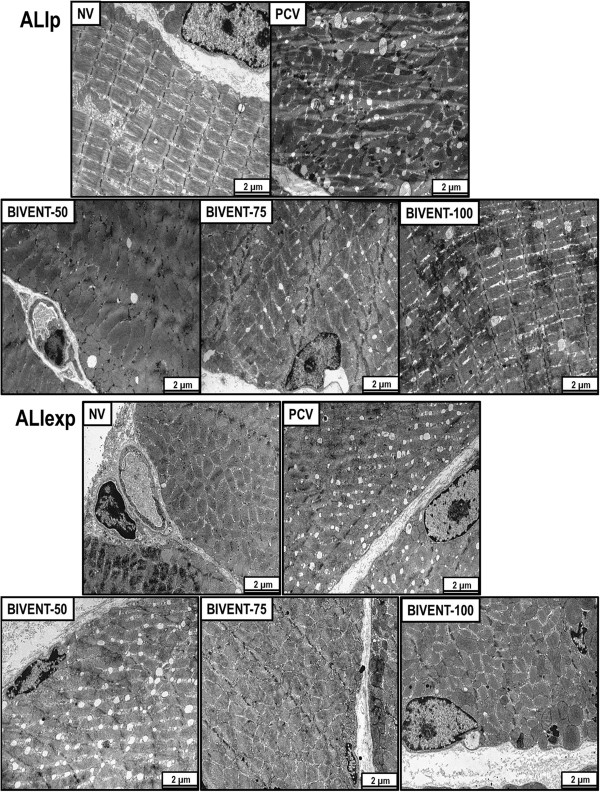
**Electron microscopy of diaphragm.** Photomicrographs are representative of data obtained from diaphragm sections of five animals per group. In ALI_p_ animals ventilated with PCV or BIVENT-100, note the presence of vacuoles. Conversely, in ALI_exp_, there were more vacuoles in BIVENT-50 compared to BIVENT-100, but a similar amount compared to PCV. ALI_exp_: extrapulmonary acute lung injury; ALI_p_: pulmonary acute lung injury; BIVENT: biphasic positive airway pressure at different rates of time-cycled controlled breaths: 100, 75 and 50 breaths/min; NV: nonventilated; PCV: pressure-controlled ventilation.

The semiquantitative analysis of lung and diaphragm electron microscopy is shown in Table 6 and Figures 6 and 7. In the ALI_p_ group, BIVENT-75 and BIVENT-50 resulted in reduced damage to alveolar capillary membranes, type II epithelial cells and endothelial cells compared to PCV. In ALI_exp_, BIVENT-75 reduced type II epithelial and endothelial cell damage, and BIVENT-50 reduced alveolar capillary membrane and type II epithelial cell damage, compared to PCV. In ALI_p_, mechanical ventilation led to myofibril damage with Z-disk edema, which was greater under PCV and BIVENT-100 than with other ventilator strategies. Mitochondrial injury of the diaphragm was less pronounced with BIVENT-50 than with BIVENT-100. Vacuoles were more abundant under PCV than under NV. BIVENT-50 resulted in a lower number of vacuoles and less diaphragm damage than other ventilator strategies. In the ALI_exp_ group, Z-disk edema was more pronounced during PCV and BIVENT-50 than during NV. Mitochondrial injury was more intense under PCV than under NV, but there were no differences in mitochondrial injury among different ventilator strategies. BIVENT-100 and BIVENT-75 resulted in fewer vacuoles and less intermyofibril space than were caused by PCV.

**Table 6:** **Semiquantitative analysis of lung and diaphragm electron microscopy**^**a**^

**Groups**		**Lung**			**Diaphragm**		
		**Alveolar capillary membrane**	**Type II epithelial cells**	**Endothelial cells**	**Abnormal myofibril with Z-disk edema**	**Mitochondrial injury**	**Miscellaneous**
ALI_p_	NV	3 (2.5-3)	3 (3-4)	2 (1.5-2)	1 (1-1.5)	2 (1-1.5)	1 (1-2)
	PCV	4 (3.5-4)	3 (3-4)	3 (2.5-3.5)	3 (3-4)^b^	2 (2-2.5)	4 (3-4)^b^
	BIVENT-100	2 (2-3)	3 (2.5-3)	2 (2-2.5)	3 (2.5-4)^b^	3 (2.5-3)^b^	3 (3-3.5)
	BIVENT-75	2 (1-2)^c^	2 (1.5-2)^b,c^	2 (1.5-2)^c^	2 (2-2.5)	2 (1.5-2)	2 (1.5-2.5)
	BIVENT-50	2 (2-2)^c^	2 (1-2)^b,c^	2 (1.5-2)^c^	2 (1-2)	1 (1-2)^d^	1 (1-1.5)^c,d^
ALI_exp_	NV	3 (2.5-3)	3 (2-3.5)	3 (3-4)	1 (1-1.5)	1 (1-1.5)	2 (1.5-2)
	PCV	3 (2.5-4)	3 (3-3.5)	3 (3-3.5)	3 (2.5-3)^b^	3 (2-3)^b^	4 (3.5-4)
	BIVENT-100	2 (2-3.5)	2 (2-3)	2 (2-3)	2 (1.5-2)	2 (1-2)	1 (1-1.5)^c^
	BIVENT-75	2 (2-2)	2 (2-2)^c^	2 (1.5-2)^b,c^	2 (1.5-2)	2 (1.5-2)	1 (1-1.5)^c^
	BIVENT-50	2 (1-2) #	2 (2-2)^c^	2 (1.5-2.5)	3 (2-3)^b^	2 (2-3)	3 (2.5-3)

^a^ALI_exp_: extrapulmonary acute lung injury; ALI_p_: pulmonary acute lung injury; BIVENT: biphasic positive airway pressure at different rates of time-cycled controlled breaths: 100, 75 and 50 breaths/min; NV: nonventilated; PCV: pressure-controlled ventilation. Values are medians and IQRs of five animals in each group. A five-point semiquantitative severity-based scoring system was used. Pathological findings were graded as percentages of examined tissue: 0 = normal lung parenchyma, 1 = 1% to 25%, 2 = 26% to 50%, 3 = 51% to 75% and 4 = 76% to 100%. ^b^NV, ^c^PCV, ^d^BIVENT-100 (*P* < 0.05).

**Figure 8:**
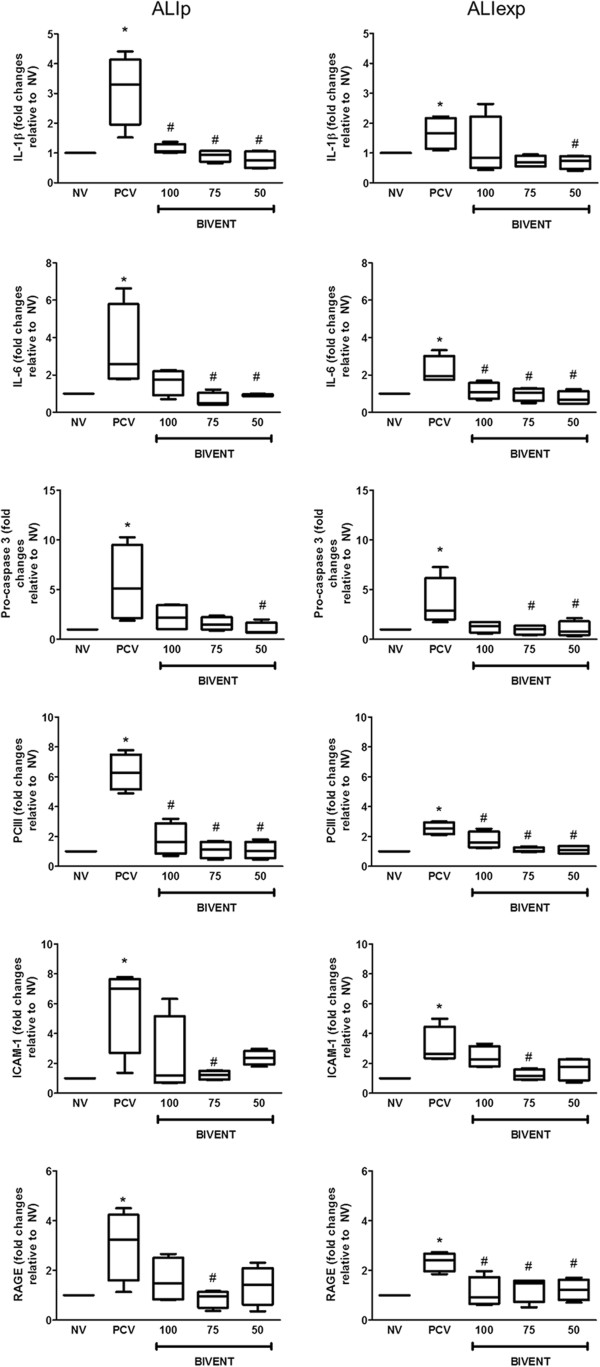
**Expression of biological markers.** Real-time polymerase chain reaction analysis of biological markers associated with inflammation (interleukin β (IL-β) and IL-6), apoptosis (procaspase 3), fibrogenesis (pro-collagen type III, PCIII) and damage inflicted upon the endothelium (intercellular adhesion molecule 1 (ICAM-1)) and alveolar type I epithelial cells (receptor for advanced glycation end product, RAGE). Relative gene expression was calculated as a ratio of the average gene expression levels compared with the reference gene (*36B4*) and expressed as fold change relative to NV (nonventilated). ALI_exp_: extrapulmonary acute lung injury; ALI_p_: pulmonary acute lung injury; BIVENT: biphasic positive airway pressure at different rates of time-cycled controlled breaths: 100, 75 and 50 breaths/min; NV: nonventilated; P2 = type II epithelial cell; PCV: pressure-controlled ventilation. Boxplots show medians and IQRs of five rats in each group. ^*^P < 0.05 vs. NV, ^#^*P* < 0.05 vs. PCV.

In both ALI models, BIVENT decreased the gene expression of interleukin 6 (IL-6), IL-1β, procaspase 3, PC_III_, ICAM-1 and RAGE in lung tissue compared to PCV (Figure 8). In ALI_p_, gene expression of IL-6 and PC_III_ was lower in all BIVENT groups compared to PCV. IL-6, ICAM-1 and RAGE mRNA was reduced in BIVENT-75, and gene expression of procaspase 3 was lower in BIVENT-50 compared to PCV. In ALI_exp_, gene expression of IL-6, PC_III_ and RAGE was lower in all BIVENT groups compared to PCV. Procaspase 3 gene expression was reduced in BIVENT-75 and BIVENT-50 compared to PCV. Expression of ICAM-1 and IL-1β mRNA was lower in BIVENT-75 and BIVENT-50, respectively, compared to PCV.

## Discussion

In the present study, we found that BIVENT promoted a more pronounced reduction in markers of inflammation, apoptosis and fibrogenesis, as well as less epithelial and endothelial cell damage, in rat models of ALI_p_ and ALI_exp_ compared to PCV. Conversely, the rate of spontaneous and assisted breaths during BIVENT led to etiology-associated levels of atelectasis and diaphragmatic injury. In ALI_p_, alveolar collapse increased during BIVENT-100 but decreased during BIVENT-50 compared to PCV, and there was less diaphragmatic injury during BIVENT-50. In ALI_exp_, alveolar collapse during BIVENT-100 and BIVENT-75 was comparable to PCV but was decreased during BIVENT-50 compared to PCV, and diaphragmatic injury increased during BIVENT-50.

To the best of our knowledge, no previous experimental study has investigated the biological impact of different rates of time-cycled control breaths during BIVENT on lung morphology, inflammation, apoptosis, fibrogenesis, epithelial and endothelial cell damage and diaphragmatic damage in ALI_p_ and ALI_exp_. In fact, PCV has been compared with BIVENT combined with pressure support at a constant rate of time-cycled control breaths [4], as well as with pressure support alone [14], but those studies did not address the etiology of ALI. In clinical practice, ALI_p_ and ALI_exp_ can overlap and their distinction is not always easy. However, the use of these two models of mild ALI might improve understanding of the mechanisms of VALI during assisted ventilation.

In our ALI_p_ and ALI_exp_ models, gene expression of inflammatory mediators, apoptosis, fibrogenesis and biochemical markers of epithelial and endothelial cell injury decreased during BIVENT compared to PCV, but did not differ between varying rates of time-cycled control breaths. BIVENT-75 and BIVENT-50 reduced ultrastructural damage to the alveolar capillary membrane and to type II epithelial and endothelial cells. Furthermore, BIVENT was associated with a reduction in gene expression of markers of endothelial and epithelial cell damage. Thus, our results suggest that the presence of spontaneous and assisted breaths is sufficient to minimize VALI compared to PCV, regardless of the level of inspiratory effort. This indicates that the reduction of atelectasis *per se* cannot explain the reduction in VALI observed with BIVENT. Accordingly, it is conceivable that other mechanisms play a role, such as more homogeneous distribution of ventilation and regional pleural pressure, as well as redistribution of perfusion [15-17].

We observed that, compared to modes with spontaneous breathing activity, PCV induced damage to the diaphragm. This finding is consistent with reports of several studies that have shown less diaphragmatic injury during assisted ventilation [18,19]. Sassoon *et al*. showed that partial respiratory muscle activation reduces muscle dysfunction in other ALI models [20]. Nevertheless, BIVENT-50 was associated with increased diaphragm injury in ALI_exp_, as evidenced by augmented vacuolization, but not in ALI_p_. A possible explanation is that the amount of muscle work during spontaneous breath cycles was relatively low in animals with ALI_exp_ that were ventilated with BIVENT-50, favoring diaphragmatic dysfunction. However, not only the amount of inspiratory effort but also RR *per se* may affect diaphragmatic injury. These data could have a potential impact on further investigations into this specific issue and highlight the importance of monitoring and evaluating RR during assisted ventilation. Our results suggest that controlled breaths during BIVENT should be cautiously reduced in ALI_exp_ to minimize diaphragmatic injury.

Pulmonary and extrapulmonary mild ALI were induced by administering *E. coli* LPS intratracheally and intraperitoneally, respectively. Both models cause similar deterioration in oxygenation, lung mechanics and alveolar collapse [9,21]. The LPS model reproduces some of the main features of ALI, such as histological tissue injury, alteration of the alveolar capillary barrier, inflammation and pulmonary dysfunction [22]. Direct lung injury (ALI_p_) primarily affects the alveolar epithelium, with damage occurring mainly in the intra-alveolar space, with alveolar flooding and areas of consolidation [9,21]. In indirect lung injury (ALI_exp_), endothelial cells are the first target of damage, with a subsequent increase in vascular permeability. Thus, the main pathologic alteration due to an indirect insult may be microvessel congestion and interstitial edema, with relative sparing of intra-alveolar spaces [9]. In view of these facts, we hypothesized that BIVENT would be more effective to reopen atelectatic lung regions (thus resulting in less VALI) in ALI_exp_ as compared to ALI_p_.

In line with current recommendations [23], we used protective mechanical ventilation with the same driving pressure to achieve a low V_t_ (6 ml/kg) during both PCV and BIVENT. The level of PEEP was set at 5 cmH_2_O because previous observations from our group suggested that higher levels may lead to hyperinflation and lung injury in these models of ALI in rats [10,21]. Unlike other types of biphasic CPAP ventilation, BIVENT allows spontaneous breaths not only during low levels of CPAP but also during high levels. Thus, ineffective breaths are avoided during the high level of CPAP. During BIVENT, inspiratory time was kept constant while changing the rate of time-cycled breaths to allow more spontaneous breaths during the low level of CPAP. The choice of ventilation settings of BIVENT was guided by our clinical experience with ARDS patients. Accordingly, we used a RR yielding full support (100%), that is, controlled mechanical ventilation, as well as half (50%) of that rate. To avoid excessive inspiratory effort and muscular fatigue, we did not use lower RRs. To minimize asynchrony, no pressure support was used during spontaneous breaths. Blood gas analysis was performed with FiO_2_ = 1.0 to avoid possible confounding effects of ventilation/perfusion mismatch in the interpretation of the gas exchange data [24]. However, this study was conducted with FiO_2_ = 0.40 to avoid possible iatrogenic effects on the lung parenchyma induced by high concentrations of oxygen [25]. Because pulmonary histology was evaluated at comparable airway pressures, lung morphometry changes mainly reflect the effects of different modes of mechanical ventilation.

Arterial blood gases were analyzed separately at baseline ZEEP and at end (PEEP = 5 cmH_2_O) in each ALI group. The dramatic recovery in oxygenation over one hour of mechanical ventilation may suggest that the hypoxemia is a consequence of atelectasis. Hypoxemia would undoubtedly occur in rodents subjected to anesthesia, surgery and mechanical ventilation with ZEEP, which favors the use of recruitment maneuvers (RMs). However, we previously observed that RMs resulted in greater type III procollagen mRNA expression in ALI_p_ than in ALI_exp_[14], and thus we avoided such maneuvers in the present study. Moreover, our goal was to investigate the role of different amount of assisted spontaneous breaths on lung injury, taking into account all the limitations of the experimental setting we used.

We found that, in both ALI_p_ and ALI_exp_, the decrease in the rate of time-cycled control breaths yielded an increase in aeration and a reduction in alveolar collapse. However, in ALI_p_, we observed an increase in alveolar collapse during BIVENT-100 compared to PCV, without impairment of gas exchange.

The main determinant of alveolar recruitment is the P_L_ achieved at end inspiration and end expiration [26]. Although the inspiratory airway and P_L_ are closely related during controlled mechanical ventilation, they can be partially dissociated during assisted ventilation, owing to respiratory muscle activation. Therefore, we measured P_es_ as an estimate of the inspiratory effort during BIVENT. The total PTP did not differ between BIVENT groups, whereas PTP during spontaneous breaths at P_low_ was increased in BIVENT-50 compared to other groups. Respiratory drive, as assessed by P_0.1_, was higher during BIVENT-50 compared to BIVENT-100 in both ALI models. However, in spontaneous breaths at P_low_, P_0.1_ was higher in BIVENT-50 compared to BIVENT-75 and BIVENT-100. The higher inspiratory effort during BIVENT-50 probably accounts for the reduced alveolar collapse in that group.

### Limitations

Our study has several limitations. (1) We used intratracheally or intraperitoneally injected *E. coli* LPS to induce mild pulmonary and extrapulmonary ALI. Thus, these data cannot be extrapolated to ALI models with different degrees of severity or to human ARDS. Nevertheless, our results improve the understanding of the mechanisms underlying VALI during assisted ventilation. (2) ALI was characterized on the basis of the presence of diffuse alveolar damage observed with light and electron microscopy as well as lung functional changes. We did not evaluate the extent of alveolar edema using the wet-to-dry ratio and the level of protein in bronchoalveolar lavage fluid. (3) We investigated the effects of different ventilator strategies in ALI_p_ and ALI_exp_ and therefore did not include a control group. This was done mainly to avoid an excessive number of comparisons and because we were interested in investigating the effects of different levels of spontaneous breaths in injured lungs. Furthermore, PEEP was not individually titrated, rather, a fixed PEEP level (5 cmH_2_O) was applied to avoid the introduction of a confounding factor. (4) The study period was short (one hour); therefore, our results cannot be extrapolated to longer periods of ventilation. However, the advantage of this short duration of mechanical ventilation is that it hinders the introduction of any additional potential factors which may affect the results, such as changes in respiratory pattern and/or hemodynamic instability, fluid overload and/or excessive sedation. (5) We conducted the experiments in small animals, and results may differ in larger animals and patients. (6) Our results are based on BIVENT and cannot be generalized to other modes of assisted ventilation and/or different ventilator settings. (7) P_high_ was kept constant during BIVENT. Thus, V_t_ changed accordingly. On the other hand, when maintaining V_t_ constant, P_high_ may change. In this line, changes in both V_t_ and P_high_ may yield VALI. However, V_t_ of mechanically controlled breaths was comparable among the different rates of time-cycled controlled breaths. (8) We did not measure inflammatory mediators in blood or distal organs. (9) We avoided a formal evaluation of asynchrony events, because we did not record the electrical activity of the diaphragm. Nevertheless, we cannot rule out an effect of subject–ventilator asynchrony on lung injury outcomes, but any such effect would likely be minor, since spontaneous breathing activity was associated with less lung injury than controlled mechanical ventilation. (10) Ultrastructural damage to the diaphragm was evaluated by semiquantitative analysis. Further studies are required to investigate functional activity and biochemical injury of the diaphragm during longer periods of mechanical ventilation.

## Conclusions

In the present models of mild ALI, we found that BIVENT had lower biological impacts than PCV on lung tissue. Atelectasis and diaphragmatic injury resulting from different rates of spontaneous and assisted breaths during BIVENT were dependent on ALI etiology (pulmonary or extrapulmonary). Therefore, care should be taken when setting controlled breaths during BIVENT in ALI.

## Key messages

• In experimental models of mild pulmonary and extrapulmonary ALI, BIVENT had less biological impact than PCV on lung tissue.

• The inspiratory effort during spontaneous breaths increased during BIVENT with a rate of time-cycled control breaths of 50/min (BIVENT-50) in both ALI models.

• In ALI_p_, alveolar collapse was higher in BIVENT-100 than in PCV, but it was decreased during BIVENT-50. In ALI_exp_, however, alveolar collapse during BIVENT-100 and BIVENT-75 was comparable to PCV but decreased during BIVENT-50.

• The diaphragmatic injury response to BIVENT differed according to the rate of spontaneous and controlled breaths and to ALI etiology.

## Abbreviations

ACB: Alveolar capillary basement; ALI: Acute lung injury; ALIexp: Extrapulmonary acute lung injury; ALIp: Pulmonary acute lung injury; ARDS: Acute respiratory distress syndrome; BIVENT: Biphasic positive airway pressure; CPAP: Continuous positive airway pressure; FiO2: Fraction of inspired oxygen; ICAM-1: intracellular adhesion molecule 1; I:E: Inspiratory:expiratory ratio; MAP: Mean arterial pressure; NV: Nonventilated; P0.1: Decay in airway pressure 100 ms after start of inspiration; PaCO2: Partial pressure of arterial carbon dioxide; PaO2: Partial pressure of arterial oxygen; PCV: Pressure-controlled ventilation, PEEP, Positive end-expiratory pressure; pHa: Arterial pH; Pmean: Mean airway pressure; Ppeak: Peak airway pressure; Ppl,mean: Mean transpulmonary pressure; PSV: Pressure support ventilation; PTP: pressure–time product of the inspiratory esophageal pressure; RAGE: Receptor for advanced glycation end product; RM: Recruitment maneuver; RR: Respiratory rate; Ti/Ttot: Inspiratory to total respiratory time; VALI: Ventilator-associated lung injury; VCAM-1: Vascular cellular adhesion molecule 1; Vt: Tidal volume; ZEEP: Zero end-expiratory pressure.

## Competing interests

Dr Gama de Abreu was granted a patent on the variable pressure support ventilation mode of assisted ventilation (noisy PSV), which has been licensed to Dräger Medical AG (Lübeck, Germany). The remaining authors have not disclosed any potential conflicts of interest.

## Authors’ contributions

FS: animal preparation, performance of experimental work, analysis of the mechanical and histological data, statistical analysis and drafting of the manuscript. LM: animal preparation, performance of experimental work, preliminary analysis of the data and helped draft the manuscript. CLS: animal preparation, performance of experimental work, analysis of the mechanical data, molecular biology analysis and help with drafting of the manuscript. GPO: animal preparation, performance of experimental work and analysis of the mechanical and morphometric data. FFC: animal preparation, performance of experimental work, analysis of the mechanical and morphometric data and help with drafting of the manuscript. MMM: analysis of the molecular biology data and help with drafting of the manuscript. VLC: analysis of the histological data help with drafting of the manuscript. MGA: experimental design, writing of the manuscript and supervision and oversight of the entire project. CSNBG: animal preparation, performance of experimental work, analysis of the mechanical data, design of the experiments and writing of the manuscript. PP: design of the experiments, writing of the manuscript and supervision and oversight of entire project. PRMR: experimental design, supervision of experimental work, statistical analysis, writing of the manuscript and supervision and oversight of the entire project. All authors revised the manuscript and approved the final version.
